# Supplementary figures and images for: Development and evaluation of rapid novel isothermal amplification assays for important veterinary pathogens: Chlamydia psittaci and Chlamydia pecorum
Source: PeerJ. 2017 Sep 8;5:e3799. doi: 10.7717/peerj.3799 (PMC5592900; doi:10.7717/peerj.3799)

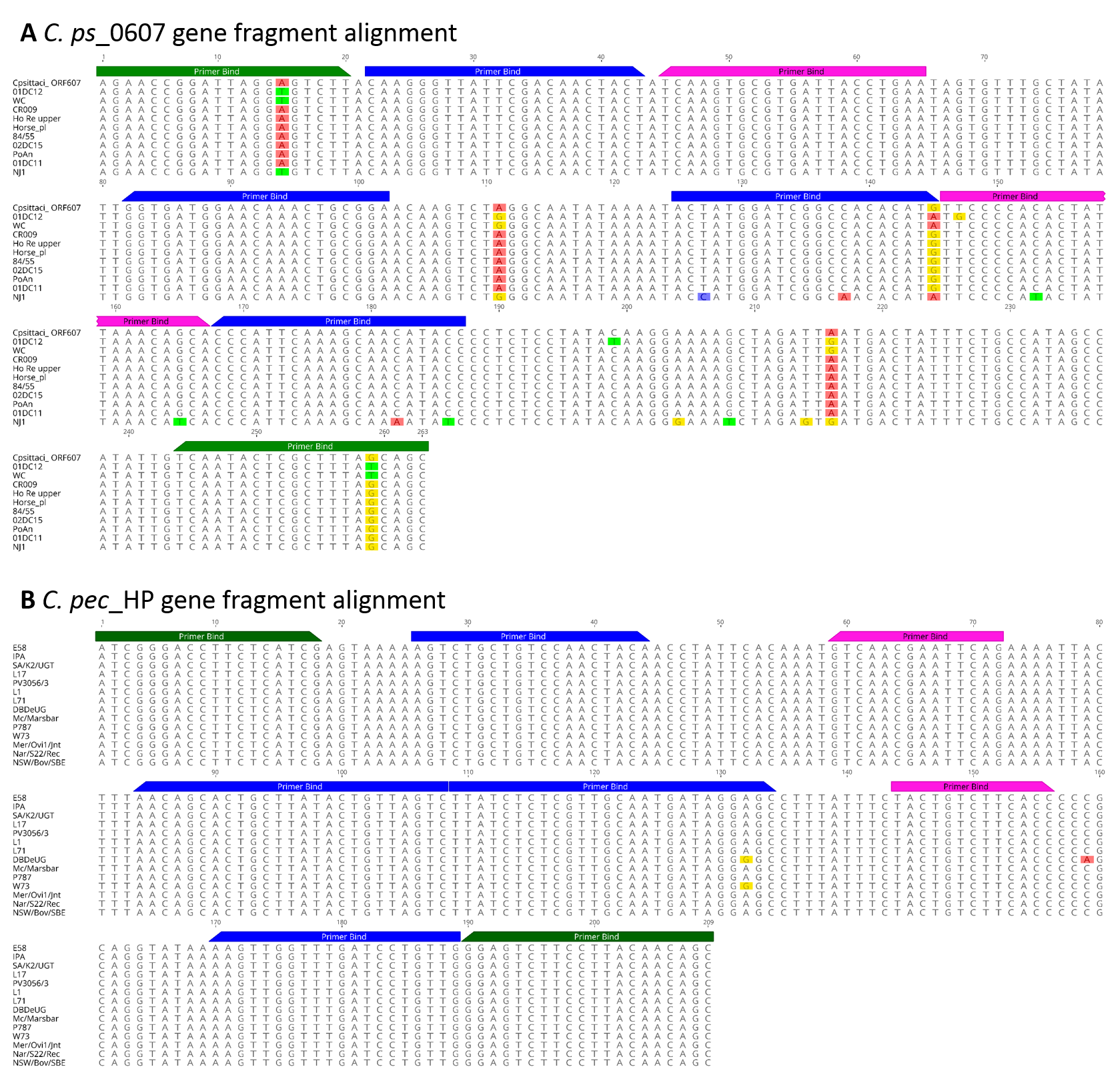

Supplement: Figure S1 — A: A 263bp fragment of the Cps_0607 gene alignment; B: A 209bp fragment of the C.pec_HP gene alignment. SNPs in the alignments are highlighted. [file peerj-05-3799-s001.png]

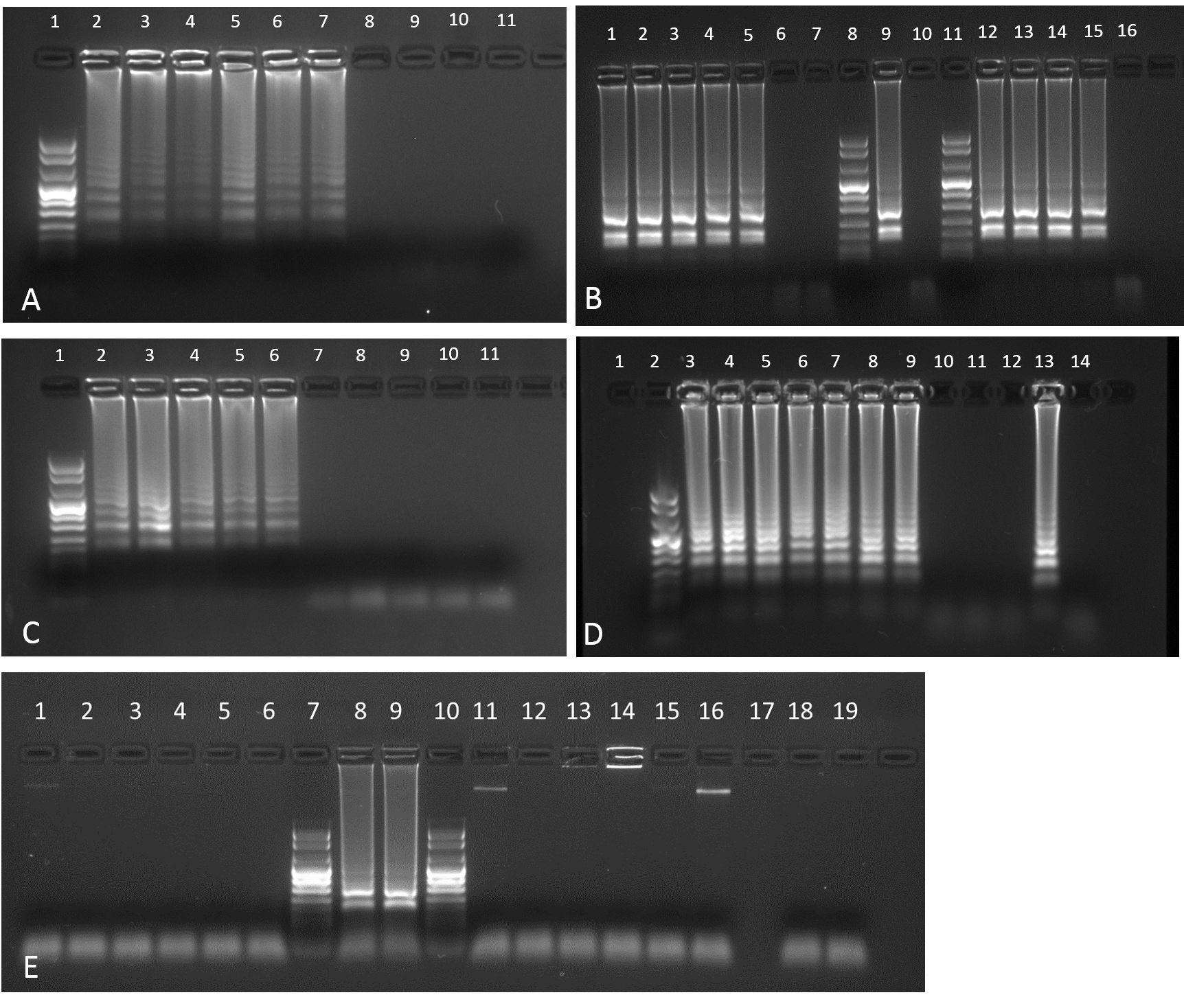

Supplement: Figure S2 — (A) C. pecorum LAMP sensitivity. In lanes 1: DNA marker VIII, 2: C. pecorum 105 genome copy number dilution, 3: 104 genome copy number dilution, 4: 103 genome copy number dilution, 5: 102 genome copy number dilution, 6: 101 genome copy number dilution, 7: 100 genome copy number dilution, 8: 10−1 genome copy number dilution, 9: 10−2 genome copy number dilution, 10: Negative (water as template), 11: Negative (mix only); (B) C. pecorum LAMP testing of clinical samples. In lanes 1: koala swab sample Chloe U, 2: Chloe R, 3: koala swab sample K61528, 4: K68199, 5: K67797, 6: K52866, 7: K60652, 8 and 11: DNA Marker VIII, 9: C. pecorum koala Marsbar strain, 10: Negative (water), 12: sheep eye swab S87, 13: Lamb1 eye swab, 14: cattle rectal swab 18R, 15: Cow brain sample, and 16: cattle rectal swab 20R; (C) C. psittaci LAMP sensitivity. In lanes 1: DNA marker VIII, 2: C. psittaci 105 genome copy number dilution, 3: 104 genome copy number dilution, 4: 103 genome copy number dilution, 5: 102 genome copy number dilution, 6: 101 genome copy number dilution, 7: 100 genome copy number dilution, 8: 10−1 genome copy number dilution, 9: 10−2 genome copy number dilution, 10: Negative (water as template), 11: Negative (mix only); (D) C. psittaci LAMP testing of clinical samples. In lanes 1: empty, 2: DNA Marker VIII, 3: Horse 14092/1, 4: Horse 10272/3, 5: Horse 13234/3, 6: Horse 11310/2, 7: Horse 12004/2, 8: Horse 12818/3, 9: Horse 11786/3, 10: Horse 11035/1, 11: Horse 13237/3, 12: Pigeon p12, 13: C. psittaci B2, 14: Negative (water as template); (E) C. pecorum LAMP specificity. In lanes 1: C. psittaci, 2: C. pneumoniae, 3: C. abortus, 4: C. suis, 5: C. trachomatis, 6: Chlamydiales positive sample (Fritchea spp.), 7 and 10: DNA Marker VIII, 8: C. pecorum koala Marsbar strain, 9: C. pecorum E58 cattle strain, 11 - 16: Gram Neg and Gram Pos bacteria, as follows: Escherichia coli, Enterococcus feacalis, Fusobacterium spp., Prevotella bivia, Staphylococcus epidermidis, Streptococcus spp., [file peerj-05-3799-s002.png]
