# Supplementary material for: Development and evaluation of rapid novel isothermal amplification assays for important veterinary pathogens: Chlamydia psittaci and Chlamydia pecorum
Source: PeerJ. 2017 Sep 8;5:e3799. doi: 10.7717/peerj.3799 (PMC5592900; doi:10.7717/peerj.3799)
Supplement: Table S1 [file peerj-05-3799-s003.pdf]

Table S1. Specificity of the *C. psittaci* and *C. pecorum* LAMP assays

Sample	Host	Site	Time to amplify	Melt	qPCR copies/ul
***C. psittaci* cultured isolates**					
CR009 cul.	Parrot	Beak	6.45	84.33	7.1x10^4
HoRe swab cul.	Human	Throat swab	5.15	84.58	1x10^6
Sz cul.	Human	Throat swab	8.15	84.63	8.6x10^3
Po An cul.	Human	Throat swab	7.15	84.58	1.2x10^6
HoRe sp.cul.	Human	Sputum	5.00	84.39	8x10^6
Fr Da cul.	Human	Throat swab	6.45	84.54	1.4x10^6
Horse_pl cul.	Horse	Placental tissue	10.30	84.53	143
CR394 cul.	Parrot	Eye	10.30	84.08	27
Fa An cul.	Human	Throat swab	7.30	84.27	2.3x10^4
H2	Human	Throat swab	8.15	84.26	4x10^4
H4	Human	Throat swab	8.30	84.32	2.1x10^6
C5	Human	Throat swab	7.45	83.57	7x10^4
***C. pecorum* cultured isolates**					
Marsbar G	Koala	UGT	13.15	83.20	1.5x10^6
A5		Bladder	12.30	83.68	2.3x10^6
A7		Eye	13.30	83.44	1.6x10^6
A11		Eye	16.15	83.35	6x10^4
A13		Cloaca	13.45	83.40	4.1x10^6
A2		UGT	11.30	83.43	9.8x10^6
PM13		Urethra	14.18	83.62	6.7x10^4
IPA	Sheep	Joint	13.15	83.33	1x10^6
W73		Feces	17.45	83.93	4.5x10^3
JP157		Feces	13.30	83.27	3.9x10^6
AB10		Placenta	20.15	83.50	1.3x10^3
E58	Cattle	Brain	13.45	83.62	1x10^6
66P130		Feces	12.15	83.38	8.6x10^6
LW679		Joint	12.00	83.48	7.5x10^6
L14		Lung	13.15	83.53	3x10^5
1920Brz	Pig	Lung	12.45	83.53	4.4x10^6
HsLuRz		Lung	13.45	83.49	1.1x10^6
L40		Lung	13.00	83.44	1.1x10^6

Specificity of the assays agaisnt other bacteria

	C. pecorum LAMP		*C. psittaci* LAMP	
**Sample**	**Time to amplify**	**Melt**	**Time to amplify**	**Melt**
***C. pecorum* M**	13.15	83.20		
***C. psittac* i CR009**			6.45	84.33
C. pneumoniae	Nil		Nil	
C. suis S45	Nil		Nil	
C. abortus	Nil		Nil	
C. trachomatis	Nil		Nil	
C.murridarum I	Nil		Nil	
C. caviae GPIC	Nil		Nil	
Uncultured Chl	Nil		Nil	
Escherichia coli	Nil		Nil	
Enterococcus f	Nil		Nil	
Fusobacterium	Nil		Nil	
Prevotella bivi	Nil		Nil	
Staphylococcus	Nil		Nil	
Streptococcus	Nil		Nil	
Human DNA	Nil		Nil	
