# Supplementary material for: Development and evaluation of rapid novel isothermal amplification assays for important veterinary pathogens: Chlamydia psittaci and Chlamydia pecorum
Source: PeerJ. 2017 Sep 8;5:e3799. doi: 10.7717/peerj.3799 (PMC5592900; doi:10.7717/peerj.3799)
Supplement: Table S2 [file peerj-05-3799-s004.pdf]

Table S2. *C. psittaci* LAMP testing of clinical samples

Sample	Host	Site	me to ampl	Melt	qPCR copies/ul	Cps qPCR CT
14092/1	Horse	Placenta tissue	7.45	83.77	5.6x10^4	29.10
13590/2	Horse	Placenta swab	16.45	83.92	BDL (3)	39.72
12934/1	Horse	Nasal swab	0.00	0.00	BDL (4)	37.95
11310/2	Horse	Foetus swab	26.45	83.63	22	37.67
10271/3	Horse	Foetus swab	13.15	82.75	56	31.37
10271/2		Foetus swab	16.45	83.00	326	30.01
11035/1	Horse	Placental swab	29.15	84.25	0	40.94
10272/2	Horse	Foetus swab	20.00	83.99	293	32.51
10272/3		Placental swab	11.15	82.90	636	29.83
11184/1	Horse	Placental swab	0.00	83.05	BDL(5)	Und
12159/2	Horse	Foetus swab	0.00	0.00	0	Und
13237/3	Horse	Lung Foetus swab	0.00	0.00	0	Und
13436/3	Horse	Placental swab	22.00	83.14	BDL(5)	39.51
13022/1	Horse	Foetal tissue	0.00	0.00	0	Und
13114/1	Horse	Foetus swab	0.00	0.00	0	Und
p11	Pigeon	Liver	0.00	0.00	0	
p13	Pigeon	Liver	0.00	0.00	0	
p12	Pigeon	Liver	0.00	84.04	BDL (4)	
13234/2	Horse	Foetus swab	25.15	83.90	BDL (3)	39.63
13234/3		Foetus swab	9.45	83.65	1092	30.44
14046/2	Horse	Foetus swab	11.15	83.95	590	34.1
11786/3	Horse	Placental swab	12.15	84.35	317	36.72
11796/2	Horse	Foetus swab	12.15	84.15	347	34.39
12004/2	Horse	Rectal Foetus swab	15.15	83.28	210	33.85
12818/3	Horse	Lung Foetus swab	11.15	84.16	5788	29.03
10652/2	Horse	Foetus swab	0.00	83.13	BDL (4)	36.99

Legend

(pink)	disagree
(green)	agree
(grey box)	agree - negative
