# Supplementary material for: Development and evaluation of rapid novel isothermal amplification assays for important veterinary pathogens: Chlamydia psittaci and Chlamydia pecorum
Source: PeerJ. 2017 Sep 8;5:e3799. doi: 10.7717/peerj.3799 (PMC5592900; doi:10.7717/peerj.3799)
Supplement: Table S3 [file peerj-05-3799-s005.pdf]

Table S3. *C. pecorum* LAMP testing of clinical samples

C. pecorum koala clinical samples

Sample	Host	Site	Time to amplify	Melt	qPCR copies/ul	LAMP TC
Chloe U	Koala	UGT	14.00	83.15	3.5x10^4	POS
Chloe R		Rectal	13.45	83.20	6.7x10^4	POS
Koala R		Rectal	20.15	83.44	4x10^3	
Kall		UGT	13.00	83.73	BDL (14)	
Kaylum		UGT	21.00	83.68	NEG	
Anastasia		Eye	0.00	0.00	NEG	NEG
K17 Eye		Eye	25.30	83.25	35	POS
k17 clo		Cloaca	23.30	83.14	140	
k52		Eye	0.00	0.00	1x10^3	NEG
k52 c		Cloaca	25.30	82.90	1.8x10^4	POS
k20 c		Cloaca	17.45	83.20	2.5x10^4	POS
K61185		Eye	24.15	82.89	1.4x10^3	POS
K61310		Eye	20.15	83.54	4.7x10^3	POS
K61254		Eye	27.15	82.90	171	POS
K61447		Eye	23.15	83.00	248	
K61452		Eye	27.00	82.86	369	
K61053		Eye	25.45	82.23	41	POS
K61832		Eye	24.15	82.88	BDL (14)	NEG
K61167		Eye	29.00	82.72	BDL (12)	NEG
K61818		Eye	0.00	0.00	NEG	NEG
K61815		Eye	0.00	0.00	NEG	
K61169		Eye	18.45	83.57	1.6X10^3	POS
K61808		Eye	0.00	0.00	NEG	NEG
K61528		Eye	26.30	82.78	1.6X10^3	POS
K68199		Eye	27.15	83.19	129	POS
K67797		Eye	19.00	83.09	114	POS
K52866		Eye	0.00	0.00	NEG	NEG
K60652		Eye	0.00	0.00	NEG	NEG
K62402		Eye	24.00	82.51	BDL (11)	POS
K67556		UGT	21.45	83.00	5.0x10^3	
K13207		UGT	24.00	82.85	422	
R1-5		UGT	17.00	83.34	1.2x10^4	
R1-7		Eye	19.45	83.00	1.6X10^3	
R15-17		Cloaca	23.30	83.14	1X10^3	
s50	Sheep	Eye	0.00	0.00	NEG	
s50 r		Rectal	0.00	0.00	BDL (35)	
S87		Eye	26.00	83.57	1.1x10^3	POS
S88		Rectal	0.00	0.00	NEG	
S57		Rectal	26.30	83.87	1.1x10^3	POS
S47		Eye	0.00	0.00	NEG	NEG
Lamb1		Eye	27	83.14	BDL (14)	POS
Lamb1		Joint	0.00	0.00	NEG	NEG
A6		Fecal	38.60	82.99	300	
A9		Eye	35.00	83.24	68	
B2		Eye	25.45	83.45	286	
B3		Fecal	0.00	83.12	NEG	
B4		Eye	35.40	83.40	BDL (5)	
B5		Fecal	0.00	0.00	NEG	
R106		Rectal	0.00	82.83	NEG	
Sheep 17		Rectal	25.30	83.50	127.00	POS
1R	Cattle	Rectal	0.00	0.00	NEG	NEG
5R		Rectal	0.00	0.00	NEG	NEG
20R		Rectal	0.00	0.00	NEG	NEG
2R		Rectal	0.00	0.00	NEG	NEG
7R		Rectal	0.00	0.00	NEG	NEG
11R		Rectal	0.00	0.00	NEG	NEG
14R		Rectal	0.00	0.00	NEG	
18R		Rectal	25.20	83.57	NEG	POS
20aR		Rectal	0.00	0.00	NEG	
Cow		Brain	25.30	83.83	110	POS
Cow65		Brain	18.30	83.43	1.1x10^3	
Cow65		Heart	28.30	83.77	1x10^3	
Cow31		Kidney	0.00	0.00	NEG	

Legend	
(pink)	disagree
(green)	agree
(grey, boxed)	agree - negative
